# Clinical perspectives of isoniazid-induced liver injury^☆^

**DOI:** 10.1016/j.livres.2021.02.001

**Published:** 2021-02-11

**Authors:** Saifei Lei, Ruizhi Gu, Xiaochao Ma

**Affiliations:** Center for Pharmacogenetics, Department of Pharmaceutical Sciences, School of Pharmacy, University of Pittsburgh, Pittsburgh, PA, USA

**Keywords:** Hepatotoxicity, Liver injury, Isoniazid (INH), Tuberculosis (TB)

## Abstract

Isoniazid (INH) is a synthetic anti-mycobacterial agent used to treat active or latent tuberculosis (TB). INH has been in clinical use for nearly 70 years and remains broadly utilized at the front line of anti-TB treatment. However, the potential for liver damage and even fulminant liver failure during INH-based TB treatment presents a major challenge for TB control programs worldwide. In this review, we discuss the hepatotoxic effects of INH and provide an overview of the mechanisms and their applications in prediction and prevention of INH hepatotoxicity in clinical practice.

## 1 Introduction

Tuberculosis (TB) remains the world’s 10th leading cause of death and the single greatest cause of death from an infectious agent.^1^ An estimated one-fourth of the world’s population is infected with *Mycobacterium tuberculosis*, with an estimated 10 million new cases and 1.5 million deaths reported in 2018.^1^ Isoniazid (INH) is arguably among the most clinically successful and extensively studied TB drugs ever to have been developed. It is a bactericide that inhibits the synthesis of mycolic acids in the bacterial cell wall, and it is effective against intracellular and extracellular organisms.^2^ INH has been approved for inclusion in combination therapies for active infection and has also been approved as a prophylactic monotherapy to prevent disease in individuals with an asymptomatic or latent tuberculosis infection (LTBI).^3^

Despite INH’s proven and robust efficacy, it has long been recognized as hepatotoxic and can cause liver failure.^4^, ^5^, ^6^, ^7^, ^8^ In spite of this long-standing awareness and extensive studies, the underlying mechanisms of INH hepatotoxicity remain poorly understood. In addition, the predictive measures for those most at risk of INH hepatotoxicity are sorely lacking. The present review summarizes clinical insights and perspectives into INH hepatotoxicity, including symptoms, incidence, risk factors, mechanisms, and management. Literatures were searched through PubMed and Google Scholar with the keywords “INH”, “hepatotoxicity”, “liver injury”, and “clinic”. Literatures were sorted by “best match” and selected by clinical relevance and reliability.

## 2 Symptoms of INH-induced liver injury

Various agents can result in hepatocyte or bile duct injury, or both, with a pattern that is hepatocellular, cholestatic, or mixed.^9^ INH-induced hepatotoxicity manifests mainly as hepatocellular necrosis.^5^ Medications can cause liver injury in a predictable time- and dose-dependent manner (*e.g.*, high doses of acetaminophen), whereas others such as INH do so more unpredictably or in an “idiosyncratic” manner. INH-induced liver injury typically occurs within weeks to months rather than days to weeks of onset.^5^ About 60% of the incidence of INH hepatotoxicity in the United States Public Health Service (USPHS) study occurred in the first 3 months of treatment, and 80% of the incidence occurred in the first 6 months.^7^^,^^8^^,^^10^ A retrospective case fatality review reported a median interval of 16 weeks from treatment initiation to symptom onset.^11^

While some individuals may be asymptomatic, others may experience symptomatic hepatotoxicity.^5^^,^^12^^,^^13^ The asymptomatic patients exhibit up to a three-fold increase over upper limit of normal range (ULN) of serum alanine transaminase (ALT) and aspartate transaminase (AST). Most cases of INH hepatotoxicity are mild and typically resolve despite continued therapy with INH. However, a small number of patients taking INH develop severe hepatitis that may progress to liver failure. INH-treated patients who are severely affected may manifest few symptoms until insidious and potentially lethal liver damage has occurred. Abdominal pain, nausea, and vomiting are observed in 50–75% of patients with severe hepatotoxicity, fever is noted in 10% and rash in 5% of patients, and dark urine, overt jaundice, and clay-colored stools are late signs of clinical worsening.^5^^,^^11^^,^^12^^,^^14^ Clinical symptoms of liver dysfunction, such as encephalopathy or jaundice, as well as the presence of severe hepatitis with aminotransferase levels >10-fold ULN are associated with a poor prognosis.^14^

## 3 Incidence of INH-induced liver injury

INH hepatotoxicity is a common complication of anti-TB therapy, ranging in severity from a transient, low-grade asymptomatic elevation of serum transaminases to fulminant hepatic failure necessitating liver transplantation.^5^^,^^15^^,^^16^ Numerous surveillance studies have assessed the overall rates of INH-induced hepatotoxicity, however, the drug-induced liver injury (DILI) Network has recently indicated that the true incidence of INH-induced liver injury is largely under-reported in the United States, and it is the second-ranking drug that causes liver injury in spite of under-reporting.^17^

A surveillance study by the USPHS of 14,000 INH-treated individuals determined a 1% overall rate (which comprises approximately 10% of patients with mild transaminase elevations) of significant, probable INH-related hepatitis.^7^ A subsequent report by the International Union Against Tuberculosis (IUAT) utilized passive detection and determined a 0.5% overall rate of hepatitis in patients receiving up to 12 months of INH, versus 0.1% receiving placebo.^10^ In a Meta-analysis of patients receiving combination therapies that include INH but not rifampicin (RIF), the incidence of hepatotoxic effects was around 1.6%; the corresponding value for regimens containing both INH and RIF was 2.5%.^18^ Death resulting from INH when used for treatment of LTBI is rare (an incidence of around 0.057%) and occurs even less frequently if proper monitoring of liver function guidelines is followed.^19^ A review based on data from the United States Food and Drug Administration (FDA) estimated 23.2 INH-associated hepatitis deaths per 100,000 patients receiving INH based prophylactic therapy.^20^

## 4 Risk factors associated with INH hepatotoxicity

### 4.1 Age

Most cases of INH hepatotoxicity are associated with age, presumably reflecting aging-related changes in liver metabolism, and susceptibility to INH-induced hepatitis and subsequent death appears to increase dramatically with advancing age.^7^^,^^8^^,^^21^^,^^22^ The Seattle-King County-based study of INH hepatotoxicity reported that the incidence of symptomatic transaminase elevation ranged from 0 in those less than 14 years of age to 0.28% in those older than 65 years,^23^ while the San Diego County study reported a similar trend toward age-related hepatotoxicity.^24^ The Memphis observational study of hepatotoxicity from INH monotherapy during LTBI treatment reported that AST elevation >5-fold ULN ranged from 0.44% in those below 35 years of age to 2.08% for those older than 49 years, a statistically significant difference.^8^ Differences in the findings among these studies may be attributed to differences in sample size for the relevant age groups, differing definitions of hepatotoxicity, patient selection, and inability to exclude confounding causes of hepatotoxicity.^25^ The severity of INH hepatotoxicity and consequent mortality has also been reported to increase with age, with higher mortality in those over 50 years of age.^5^^,^^11^^,^^20^

### 4.2 Gender

Although it has been suggested that INH hepatotoxicity might be more common in females than in males, especially the more severe forms of hepatitis leading to liver failure and death,^26^ not all studies have reported this finding and no clear evidence indicates an overall sex-related difference in the incidence of INH hepatotoxicity. The Seattle–King County study reported a nonsignificant trend toward higher INH-related hepatotoxicity in women compared with men, although the incidence of severe hepatotoxicity was relatively low in both men and women.^23^ The USPHS study showed no overall difference between women and men in rates of probable INH hepatotoxicity.^7^ The San Diego and Memphis studies also reported no significant associations between INH hepatotoxicity and sex.^8^^,^^24^

### 4.3 Racial differences

In the aforementioned USPHS study, African-American males exhibited a diminished risk of INH related hepatitis compared to white males, but no difference was noted for women of any race.^7^ The Seattle–King County study reported a nonsignificant trend toward higher hepatotoxicity in white individuals.^23^ The Memphis study failed to find associations with INH hepatotoxicity among racial groups or demographic subgroups.^8^ Thus, the data regarding racially based risks for high-grade INH-related hepatotoxicity are inconsistent, and at present, insufficient evidence exists to consider any ethnic group as a high-risk population that warrants specific follow-up or treatment modification.^25^

### 4.4 Co-treated drugs

When INH is administered for active TB in combination with other drugs, the incidence of hepatotoxicity is greater. RIF, a first line anti-TB drug, is a human specific activator of pregnane X receptor (PXR), a xenobiotic nuclear receptor that regulates the expression of drug-metabolizing enzymes including cytochromes P450 (CYP).^27^, ^28^, ^29^ In patients receiving INH, RIF appears to promote the formation of toxic INH metabolites and potentiate INH hepatotoxicity.^18^^,^^30^ Similar to RIF, CYP inducers carbamazepine and phenobarbital also increase the risk of INH-induced liver injury.^31^^,^^32^ In addition, subjects who abuse alcohol and/or drugs have a higher risk for hepatotoxicity while taking INH.^33^^,^^34^ Ethionamide and para-aminosalicylic acid may exacerbate the toxicity of INH by interfering with its acetylation.^35^, ^36^, ^37^ Furthermore, when other hepatotoxic medications (*e.g.*, azole antifungals, methotrexate, anticonvulsants, halothane or acetaminophen) are used alongside anti-TB therapy, rates of hepatotoxic effects can increase.^38^^,^^39^

### 4.5 Pre-existing liver diseases

Not surprisingly, the hepatotoxic effect of INH becomes more evident in individuals with pre-existing liver diseases.^39^^,^^40^ Elevated baseline transaminases are an independent risk factor for INH hepatotoxicity.^8^^,^^41^ The Memphis retrospective study of INH-treated TB patients found that a baseline AST greater than the ULN was a risk factor for developing transaminase elevation greater than five times the ULN upon INH treatment.^8^ Similarly, the severity of DILI may be greater in INH-treated patients with pre-existing liver diseases,^41^ but the underlying mechanism remains unclear. Inflammatory status in pre-existing liver diseases was believed to contribute to the elevated risk of INH hepatotoxicity. Hepatocytes are more sensitive to INH toxicity when exposed to a non-toxic level of H_2_O_2_.^42^ In addition, a low dose of endotoxin lipopolysaccharide (LPS), a cellular mediator of inflammation, potentiates INH hepatotoxicity.^43^ Furthermore, the immune tolerance impaired mouse models are more sensitive to INH hepatotoxicity.^44^

### 4.6 Genetic predisposition

Genetic predisposition to INH-related hepatotoxicity is an important risk factor, but currently no clinical test for such predisposition is available.^7^^,^^45^^,^^46^ The acetylation rate is a genetic phenotype that varies from patient to patient, and acetylation is a crucial step in INH metabolism. Although early studies reported a bimodal pattern of drug acetylation in a given population,^47^^,^^48^ more recent studies have genotyped variants of N-acetyl transferase 2 (NAT2), the dominant enzyme that catalyzes the acetylation of INH, to define acetylator phenotype, and the single nucleotide polymorphisms that have been investigated vary between studies. NAT2 genotypes can be grouped into three different phenotypes: slow acetylator, intermediate acetylator, and rapid acetylator.^49^ While these phenotypes exhibit equivalent INH antimicrobial activity, it is presently controversial and unclear whether patients of the slow acetylator phenotype are more likely than rapid acetylators to manifest INH hepatotoxicity. Nevertheless, most recent studies suggest that the risk of INH hepatotoxicity increases in slow acetylators.^50^^,^^51^ It has been shown that individuals carrying NAT2 variant alleles at position 481C > T, 590G > A, 857G > A had a higher risk of INH hepatotoxicity.^52^ In addition, polymorphisms of NAT2 appear to influence its reactivity with INH and eventually INH safety.^53^

The risk factors for INH-induced liver injury have been identified among polymorphisms of drug-metabolizing enzymes other than NAT. CYP2E1 is well-known for its involvement in the formation of reactive oxidative species and bioactivation of hepatotoxins such as carbon tetrachloride and acetaminophen.^54^ Subjects homozygous for the CYP2E1 c1 (wild type) allele exhibited higher transaminase activity and a 2.5-fold increased risk of INH-related hepatotoxicity compared to those with one or more CYP2E1 c2 allele. The risk of INH hepatotoxicity increased 7-fold when CYP2E1 c1/c1 was combined with slow-acetylator status.^55^, ^56^, ^57^ However, other reports have come to different conclusions, showing no significant association of CYP2E1 genotype with anti-TB DILI,^58^^,^^59^ thus the role of CYP2E1 polymorphism in INH hepatotoxicity remains controvertible and merits further investigation.

In addition, deficiency of glutathione S-transferases (GSTs) activity due to a homozygous null genotype of either or both of the GSTM1 and GSTT1 genes can influence susceptibility to INH hepatotoxicity and appears to be associated with a high incidence of hepatotoxic effects of anti-TB therapy.^60^, ^61^, ^62^ Deficiency of superoxide dismutase (SOD) may also increase the risk of INH hepatoxicity.^63^ Furthermore, polymorphisms in the carboxylesterase 1 (CES1) gene have a possible association with INH hepatotoxicity, but the authors acknowledged the necessity for replication of the results in a larger cohort for confirmation of these possible correlations.^64^ Associations with INH hepatitis and polymorphisms in the tumor necrosis factor-alpha (TNF-α) gene and the class II major histocompatibility complex (MHC) allele HLA-DQB1∗02:01 have also been described, although the effect and sample sizes in these studies were small.^65^^,^^66^

Moreover, polymorphisms of nitric oxide synthase (NOS), BTB and CNC homology (BACH), and the small Maf basic leucine zipper protein MafK, which are involved in the antioxidative response, appear to be genetic determinants in anti-TB drug induced hepatotoxicity.^67^ Uridine 5′-diphospho glucuronosyltransferase (UGT) was also found to be associated with the susceptibility of INH hepatotoxicity.^68^^,^^69^ However, more studies are needed to confirm these findings.^70^

## 5 Mechanisms of INH-induced liver injury

The mechanisms of INH-induced liver injury have been extensively investigated in clinical and preclinical studies with perspectives from INH metabolites, INH-endobiotics interactions, oxidative stress, mitochondrial injury, and immune response.

### 5.1 Association of INH metabolites with liver injury

**Fig. 1:**
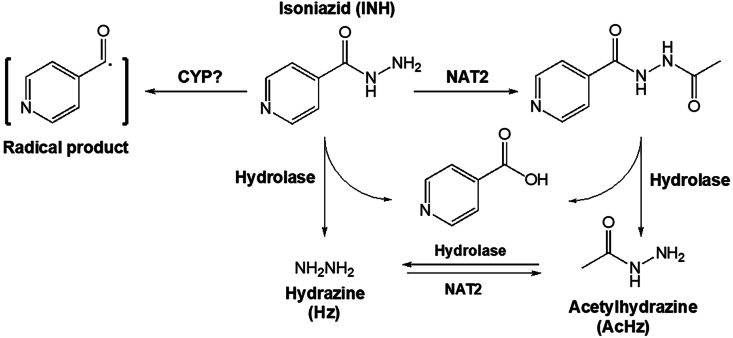
**The major metabolic pathways of INH and its association with hepatotoxicity.** Acetylation via NAT2 is a predominant pathway in INH metabolism in the liver. INH metabolites including Hz, AcHz, and isonicotinic acyl radical have been considered as a cause of INH hepatotoxicity. Abbreviations: AcHz, acetylhydrazine; CYP, cytochrome P450; Hz, hydrazine; INH, isoniazid; NAT2, N-acetyl transferase 2.

Orally administered INH is absorbed rapidly through the gastrointestinal tract and is distributed to many organs, including the liver, kidney, and brain.^71^ INH is predominantly metabolized in the liver, and three metabolites of INH have been posited to be responsible for INH-induced liver injury (Fig. 1): hydrazine (Hz), acetylhydrazine (AcHz), and a radical metabolite resulting from the bioactivation of INH itself.^72^, ^73^, ^74^, ^75^, ^76^, ^77^ The isonicotinic acyl radical can form covalent adducts to liver macromolecules and potentially trigger immune responses.^74^^,^^78^ Indeed, mass spectrometric characterization has revealed INH adducts on several murine liver proteins.^79^ However, the mechanisms for the formation of INH radical and its interaction with liver proteins remain unclear. It is also unknown whether CYPs are necessary in INH bioactivation; and if CYPs are needed, it is unknown which CYP isoform(s) is essential. CYPs have also been proposed in AcHz bioactivation,^77^ which can produce reactive metabolites and covalently bind to liver macromolecules, leading to liver damage. However, it is also unclear whether and which CYP(s) is involved in AcHz bioactivation.

In addition to isonicotinic acyl radical and AcHz, Hz has also been proposed as a cause of INH-induced liver injury.^75^^,^^76^ Hz is produced through the hydrolysis of INH or AcHz (Fig. 1). INH metabolism through these hydrolysis pathways is significantly increased in slow acetylators,^80^ especially in association with RIF co-treatment.^30^ CYP2E1 has been thought to be critical in Hz bioactivation to produce reactive derivatives/metabolites with greater toxicity, and subjects with CYP2E1 c1/c1 alleles and higher enzymatic activity have been shown to be more prone to INH hepatotoxicity.^55^^,^^56^ However, no direct evidence is available to prove the role of CYP2E1 in Hz metabolism and bioactivation.^81^ In addition, treatment with a high dose of Hz in mice failed to cause significant liver damage, especially for hepatocellular necrosis.^82^

### 5.2 INH-endobiotics interactions and their contributions to INH-induced liver injury

Chronic treatment with INH causes protoporphyrin IX (PPIX) accumulation in mouse liver.^83^ PPIX, an intermediate in the heme biosynthesis pathway, is known to be a hepatotoxin, and has been implicated in cholestasis in both mice and humans.^84^, ^85^, ^86^ INH causes PPIX accumulation in the liver through the induction of delta-aminolevulinate synthase 1 (ALAS1) and downregulation of ferrochelatase (FECH), both of which are pivotal enzymes in regulating heme biosynthesis.^83^ Rather than being caused by INH itself, PPIX accumulation is due to Hz and INH-vitamin B6 conjugate, which upregulates ALAS1 and decreases FECH, respectively.^87^ When INH is co-treated with RIF, more PPIX is accumulated in mouse liver because RIF-mediated PXR activation strongly upregulates ALAS1 expression.^88^ However, PPIX mainly contributes to cholestatic injury,^84^, ^85^, ^86^ but not hepatocellular injury, the major form of liver damage caused by INH.^5^^,^^6^

In addition to PPIX, INH can directly react with and conjugate with a number of endogenous metabolites including ketone acids, leading to the formation of hydrazones. The condensations with INH include pyruvic acid (PA) and vitamin B6, which respectively form INH-pyruvic acid (INH-PA) adduct and INH-pyridoxal (INH-PL) adduct.^89^, ^90^, ^91^ The latter conjugation of INH with vitamin B6 leads to the depletion of pyridoxal-5-phosphate in both humans and rodents.^92^^,^^93^ Five novel INH-hydrazones were identified in human urine via an LC-MS-based metabolomics approach as the condensation of INH with keto acids that are intermediates in the metabolism of leucine and/or isoleucine, lysine, tyrosine, tryptophan, and phenylalanine.^89^ INH can also react with β-nicotinamide adenine dinucleotide (NAD^+^) to form INH-NAD adduct as catalyzed by the cluster of differentiation 38, a multifunctional enzyme with NAD^+^ nucleosidase activity.^90^^,^^94^^,^^95^ However, whether these INH-endobiotics interactions play a role in INH hepatotoxicity remains to be determined.

### 5.3 Oxidative stress and mitochondrial damage in INH-induced liver injury

The association between INH hepatotoxicity and oxidative stress/reactive oxygen species (ROS) has been proposed.^96^^,^^97^ As discussed in the risk factors section above, deficiency in GST activity due to homozygous null mutations at GSTM1 and GSTT1 loci can influence susceptibility to INH hepatotoxicity. Earlier animal experiments have shown reduced levels of GSTs and other antioxidative enzymes after the administration of Hz.^98^ In addition, a murine study has established the role of microRNA-122 in oxidative stress-related liver injury by INH.^99^ Further evidence indicates that dysregulation of transcription factors that regulate glutathione synthesis and detoxification enzymes, including the transcription factor nuclear factor erythroid 2-related factor 2 (Nrf2), small Maf basic leucine zipper proteins, and the transcription factor Bach 1, may contribute to the propagation of anti-TB DILI.^67^ Moreover, reports have suggested a stronger correlation of higher severity of INH hepatotoxicity with reactive nitrite species (RNS) than with ROS; thus it is possible that peroxynitrite (ONOO^−^) generation and mitochondrial dysfunction contribute significantly to such toxicity.^100^^,^^101^

Mitochondria are an important target in DILI, as inhibition of the mitochondrial respiratory chain results in adenosine triphosphate (ATP) depletion and accumulation of ROS. An *in vitro* study found that co-exposure of hepatocytes to INH and nontoxic concentrations of the complex I inhibitors result in massive ATP depletion and cell death.^102^ The same study also found that Hz directly inhibits the activity of complex II, suggesting that underlying inhibition of complex I can potentiate INH-induced hepatocellular injury.^102^ In addition, polymorphic alleles of SOD, a mitochondrial matrix-resident protein that plays a crucial role in the detoxification of superoxide anion radicals that arise constantly during electron transport have been associated with susceptibility to INH-related hepatotoxicity.^63^^,^^103^ Furthermore, a recent study demonstrated that INH induces oxidative stress and mitochondrial dysfunction in isolated liver mitochondria, and posited that INH hepatoxicity may be mediated through an interaction with the electron transport chain, lipid peroxidation, mitochondrial membrane potential change, and cytochrome *c* extrusion, ultimately resulting in detrimental cell signaling.^104^ Moreover, a pattern of metabolic changes and steatosis consistent with mitochondrial injury was observed in comprehensive studies of INH in a panel of genetically diverse mice.^105^ However, convincing data from preclinical studies, such as development of animal models with obvious INH-induced liver injury, are lacking to prove the roles of oxidative stress and mitochondrial damage in INH hepatotoxicity.

### 5.4 Immune response in INH-induced liver injury

Involvement of immune responses in INH hepatotoxicity has been proposed for a long time. A positive lymphocyte transformation test was found in mild cases of INH-induced liver injury when patients’ lymphocytes were exposed to INH-modified proteins; in the more severe cases of liver injury, this response spread to the recognition of the parent drug.^106^^,^^107^ A further study in 2014 identified anti-INH antibodies and anti-CYP antibodies in the sera of patients with INH-induced liver injury,^78^ indicating that CYP-mediated INH metabolism produces reactive metabolite(s), which covalently binds to CYPs and other proteins in the liver and triggers immune responses. The same study identified antibodies to CYP2E1 modified by INH, CYP2E1, CYP3A4, and CYP2C9, none of which were detected in sera from INH-treated control subjects without significant liver injury.^78^ These are the most promising data showing that INH-induced liver injury is dependent on CYP-mediated INH bioactivation and immune responses. Follow-up studies are highly suggested to explore the application of anti-INH and anti-CYP antibodies in the clinic to predict and prevent INH-induced liver injury.

## 6 Management of INH-induced liver injury

While most cases of INH hepatotoxicity are mild and resolve despite continued therapy with INH, a small number of patients taking INH develop severe hepatitis that may progress to fulminant liver failure and death if INH is not stopped promptly. Biochemical tests (ALT and AST) together with the appearance of clinical symptoms (fatigue, nausea, poor appetite or jaundice) have been used to guide decisions on discontinuation of INH therapy to prevent serious liver injury.^12^ However, INH hepatotoxicity remains a serious safety concern in the clinic because of its idiosyncratic nature. In addition, no specific therapies are currently available for the treatment of INH-induced liver injury. Corticosteroids, a class of steroid hormones that have anti-inflammatory and immunosuppressive effects, are often used, but the outcomes are not convincing.^12^ Therefore, mechanism-based strategies are urgently needed for the management of INH-induced liver injury.

## 7 Summary

**Fig. 2:**
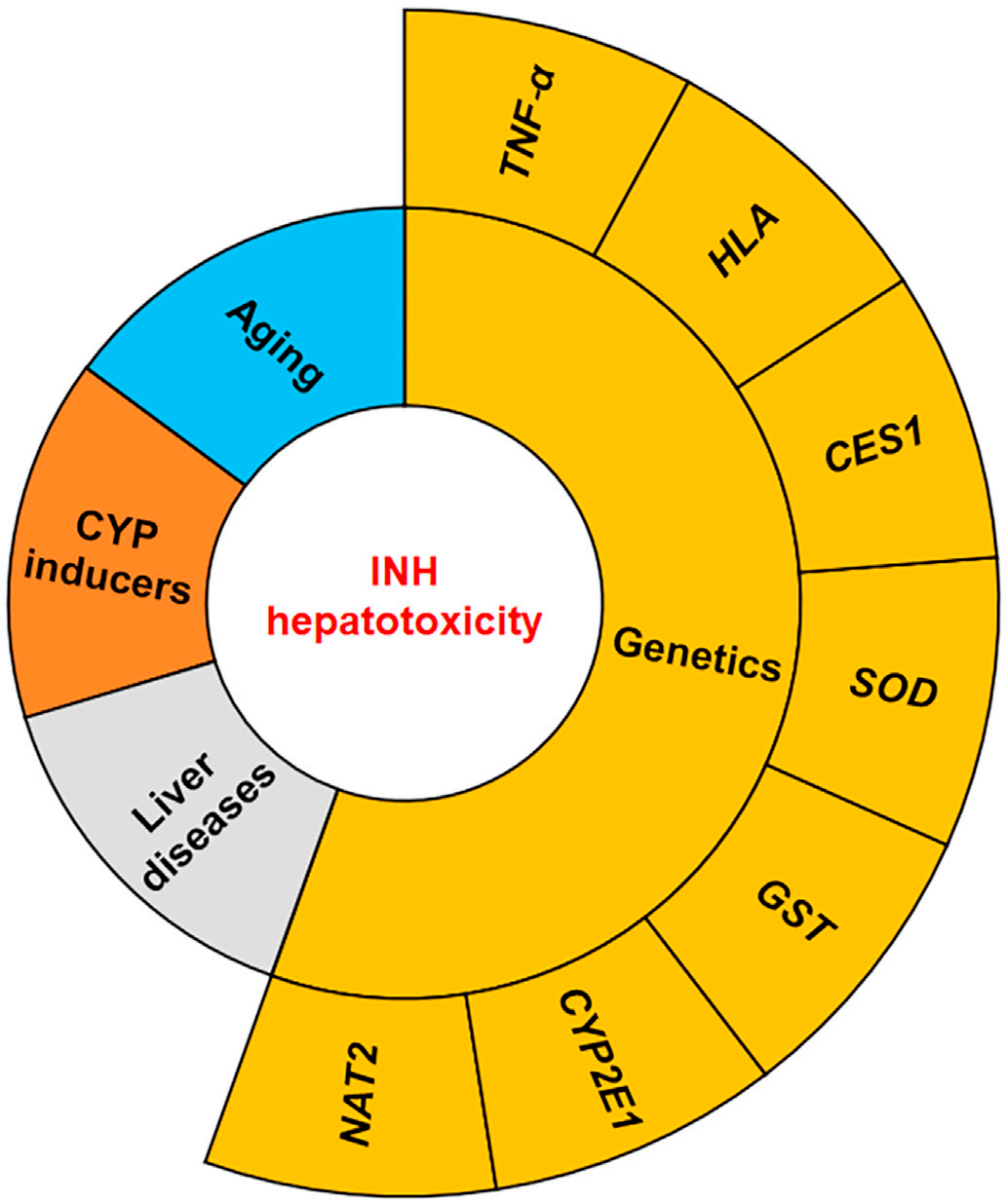
**Risk factors associated with INH hepatotoxicity.** Aging, co-treatment with drugs as CYP inducers, individuals with pre-existing liver diseases, and many genetic polymorphisms have been identified as risk factors of INH hepatotoxicity. Abbreviations: CES1, carboxylesterase 1; CYP, cytochrome P450; GST, glutathione S-transferase; HLA, human leukocyte antigen; INH, isoniazid; NAT2, N-acetyl transferase 2; SOD, superoxide dismutase; TNF-α, tumor necrosis factor-alpha.

After nearly 70 years of clinical use, INH remains a steadfast and broadly used frontline drug for TB chemotherapy and prophylaxis due to its powerful efficacy, cost-effectiveness, and usually favorable safety profile. However, INH-induced liver injury continues as a major concern in clinical practice and it remains unknown how to predict and prevent such toxicity.^5^^,^^13^^,^^20^^,^^97^ From clinical practice, multiple risk factors of INH hepatotoxicity have been identified including aging, co-treatment with drugs as CYP inducers, and individuals with pre-existing liver diseases (Fig. 2).^7^^,^^8^^,^^18^^,^^21^^,^^22^^,^^30^, ^31^, ^32^^,^^39^^,^^40^ However, the detailed mechanisms by which these factors potentiate INH-induced liver injury remain unclear. In addition, many genetic polymorphisms have been found to be associated with INH-induced liver injury (Fig. 2), but currently no clinical test for such genetic predisposition is used in clinical practice to improve the safety profile of INH.^7^^,^^45^^,^^46^

Although extensively studied, the underlying mechanisms for INH-induced hepatotoxicity remain enigmatic. This is partly due to the complexity of these mechanisms, but also because of the difficulty of distinguishing between patient-related and drug-specific factors that may determine susceptibility to INH hepatotoxicity.^96^ Multiple mechanisms of INH hepatotoxicity have been proposed, including INH metabolite-mediated toxicity, disruption of endobiotic homeostasis, oxidative stress, mitochondrial damage, and immune-mediated toxicity.^72^^,^^73^^,^^78^^,^^88^^,^^96^^,^^97^^,^^102^ However, most of these mechanisms are drawn based upon preclinical studies and are inconclusive. The identification of anti-INH antibodies and anti-CYP antibodies in the sera of human subjects with INH-induced liver injury strongly supports the role of immune responses in INH hepatotoxicity,^78^ which also sheds light on the prediction and prevention of INH hepatotoxicity, but so far no follow up report is available to show its application in the clinic.

In summary, INH hepatotoxicity remains a safety concern in clinical practice and no mechanism-based approaches are available to predict, prevent, and cure such toxicity. Further clinical and pre-clinical studies coupled with cutting-edge technologies are necessary to better elucidate and address the unanswered questions and underlying mechanisms of INH hepatotoxicity.

## Authors’ contributions

S. Lei, R. Gu, and X. Ma conducted literature search and drafted the manuscript. S. Lei and X. Ma revised the manuscript.

## Declaration of competing interest

The authors declare that they have no conflict of interest.
